# The association between mean platelet volume levels and poststroke depression

**DOI:** 10.1002/brb3.1114

**Published:** 2018-09-03

**Authors:** Huihua Qiu, Yuntao Liu, Hongfei He, Yuemin Wu, Weilei He, Guiqian Huang, Jincai He

**Affiliations:** ^1^ Department of Neurology The First Affiliated Hospital of Wenzhou Medical University Wenzhou China

**Keywords:** acute ischemic stroke, depression, mean platelet volume, risk factor

## Abstract

**Objective:**

High levels of mean platelet volume (MPV) had been found in depression subjects. We sought to examine the relationship between MPV and poststroke depression (PSD).

**Methods:**

One hundred and eighty‐five patients with acute ischemic stroke were enrolled in our study. Peripheral venous blood samples were drawn at admission and MPV levels were measured by the automated hematology analyzer. Patients with a HAMD‐17 score >7 were diagnosed as having PSD.

**Results:**

We found that 60 patients (32.4%) developed PSD, the MPV levels in PSD patients were significantly higher (9.3 ± 1.8 fl) compared to non‐PSD patients (8.5 ± 1.6 fl). High MPV levels (≥9.1 fl) were independently correlated with PSD (OR 2.762, 95% CI 1.138–6.702, *p* = 0.025).

**Conclusions:**

Patients with higher levels of MPV at admission were correlated with the development of PSD at 1 month after stroke and might be a predictor of its presence.

## 1 INTRODUCTION

Poststroke depression (PSD) is one of the most common neuropsychiatric sequelae following by stroke (Robinson, 2003). According to a meta‐analysis, approximately 31% of patients suffered from depression within five years after onset of acute stroke (Hackett & Pickles, 2014), which might negatively affect functional outcome as well as quality of life (Guajardo et al., 2015; Parikh et al., 1990). Thus, early detection of PSD is of great significance. However, the underlying mechanisms of PSD remain unclear.

Stroke is accompanied by changed platelet function, such as enhancement of platelet aggregation and activity (Grau et al., 1998; Tohgi, Suzuki, Tamura, & Kimura, 1991). As a marker of platelet function, Mean platelet volume (MPV) involves in platelet reactivity, including aggregation and release of platelet factor 4, thromboxane A2, as well as β‐thromboglobulin (Sharp et al., 1995). As a meta‐analysis comprising 24 trials demonstrated, MPV is a positively associated with incidence of myocardial infarction as well as restenosis after coronary angioplasty (Chu et al., 2010). Meanwhile, high MPV levels had been found in patients with aortic arterial stiffness, peripheral artery disease (Balta et al., 2014; Berger, Eraso, Xie, Sha, & Mohler, 2010). Previous studies reported that high MPV levels were correlated with increased risk of ischemic stroke as well as unfavorable prognosis among survivors of stroke (Bath, Algert, Chapman, & Neal, 2004; Muscari et al., 2009).

Besides, high MPV levels were also observed among patients who got depression or panic disorder (Buriachkovskaia et al., 2006; Kokacya et al., 2015). It has been established that MPV was associated with higher platelet reactivity (Bath & Butterworth, 1996; van der Loo & Martin, 1999; Yetkin, 2008), which is involved in the pathophysiological mechanisms of depression (Musselman et al., 1996; Nemeroff & Musselman, 2000).

Given the relationship between depression and MPV, it would be interesting to investigate whether MPV levels were correlated with the development of depression among patients after ischemic stroke. To date, however, no study has been conducted to examine the relationship between MPV and PSD. Hence, whether MPV levels correlate with PSD at 1 month after ischemic stroke was explored.

## 2 MATERIALS AND METHODS

### 2.1 Study population

Patients with acute ischemic stroke in the Stroke Unit of the First Affiliated Hospital of Wenzhou Medical University between October 2013 and May 2015 were admitted to our study. Patients were recruited if they: (a) were 18–80 years old; (b) were diagnosed with acute ischemic stroke occurring within one week and were confirmed by computed tomography (CT) or magnetic resonance imaging (MRI) reports; (c) were competent to consent to take part in the research. The exclusion criteria included: (a) significant acute or severe illness such as infection, heart failure and tumor; (b) patients with diseases which might affect MPV levels, such as bone marrow diseases and hypersplenism; (c) previous diagnosis of depression or other mental disorder or who had recently accepted antidepressants or antipsychotics; (d) severe aphasia or dysarthria; (e) a previous history of neurological illness including Alzheimer's disease and Parkinson's disease; (f) those who failed to have MPV levels measured at admission.

This study was conducted in accordance with the principles of the Declaration of Helsinki and approved by the Medical Ethics Committee of the First Affiliated Hospital of Wenzhou Medical University. All patients had signed an informed consent.

### 2.2 Data collection

The demographic as well as clinical characteristics of our research were obtained through standardized questionnaires interviewed by trained neurological physicians who were blind to the patients’ laboratory results at admission. Stroke etiology was classified based on TOAST criteria (Adams, 1993). The National Institutes of Health Stroke Scale (NIHSS) was used to evaluate the stroke severity on admission. The Barthel Index (BI) as well as modified Rankin Scale (mRS) were applied for assessment of functional outcome at one month after acute ischemic stroke. Besides, The Mini‐Mental State Examination (MMSE) was used for evaluation of cognition function at one month after acute ischemic stroke.

### 2.3 Definition of PSD

Depressive symptoms were evaluated by trained psychiatrists who were blind to the patients’ laboratory results using the 17‐item Hamilton Depression Scale (HAMD‐17) (Zimmerman, Martinez, Young, Chelminski, & Dalrymple, 2013). Subjects with a score >7 (Park et al., 2017; Zimmerman, et al., 2013) at one month after acute ischemic stroke were considered PSD according to DSM IV.

### 2.4 Laboratory tests

The peripheral venous blood samples were drawn on admission and collected in a calcium ethylenediaminetetra‐acetic acid tube. Complete blood count was analyzed by the automated hematology analyzer (Sysmex Company, XE‐2100, Japan) within 1 hr after sample collection. Platelet count and MPV levels were recorded. MPV levels were further divided into tertiles (≤7.7 fl, 7.8–9.0 fl, ≥9.1 fl).

### 2.5 Statistical analysis

The results were expressed as percentages for categorical variables, and continuous variables according to their normal distribution were indicated as median (interquartile range, IQR) or mean standard deviation (*SD*). Proportions were compared employing the Chi‐squared test, and continuous variables were compared by the analysis of variance (ANOVA), Mann–Whitney test, as well as Student *t* test between PSD and non‐PSD groups, as appropriate. The effect of MPV on PSD was evaluated by binary logistic regression analysis in which factors with* p < *0.05 in the univariate analysis between groups were included. In addition, variables such as hyperlipidemia, current drinking and lesion location were also included in the logistic regression analysis. Results were indicated as adjusted odds ratio (OR) (95% confidence interval, CI). Statistical analyses were used in IBM SPSS Statistics 19.0. All *p* values were two‐tailed and the significance level was set at 0.05.

## 3 RESULTS

### 3.1 Characteristics of the study population

**Figure 1:**
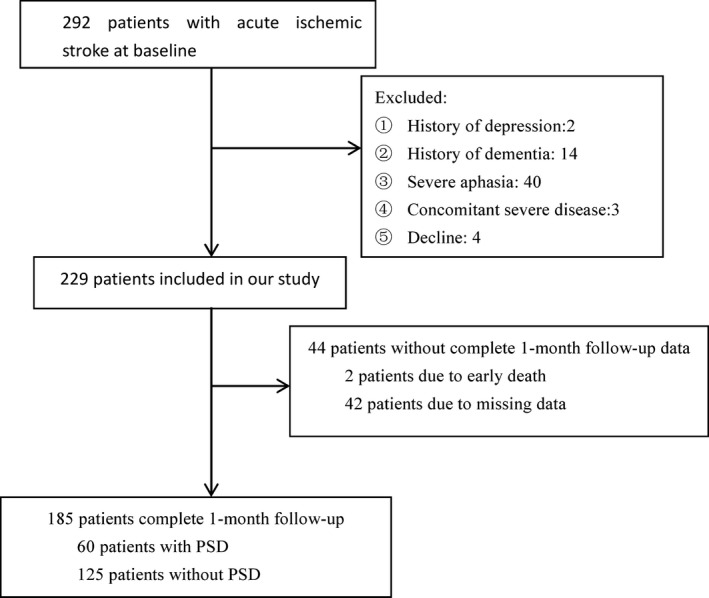
Study recruitment profile

A total of 292 acute stroke patients were screened, and 229 patients (78.4%) met the entry criteria while 63 patients (21.6%) were excluded from the study at baseline. By the time of 1‐month follow‐up, 185 patients were finally enrolled in our study (Figure 1). Compared to patients included in the study, patients excluded from the study had a higher proportion of hypertension (*p* < 0.05). No significant differences were found in terms of other baseline characteristics, such as age (*p = *0.139), proportion of female gender (*p* = 0.248), years of education (*p* = 0.462), coronary heart disease (*p* = 0.670), hyperlipidemia (*p* = 0.681), history of stroke (*p* = 0.407), current smoking (*p = *0.322), current drinking (*p* = 0.362), lesion location (*p* = 0.383), and stroke etiology (*p* = 0.438).

In the study population, 60 (32.4%) were diagnosed as having PSD. Meanwhile, the average MPV levels for all patients were 8.7 ± 1.7 fl; the levels of MPV in PSD group were significantly higher than that in non‐PSD group (9.3 ± 1.8 fl vs. 8.5 ± 1.6 fl; *F* = 9.64, *p* = 0.002). Furthermore, a more severe stroke, worse functional outcome as well as worse cognitive function were observed in PSD patients (all *p < *0.05). Moreover, the proportion of female gender was higher in PSD than non‐PSD (*p* < 0.05). No association was found between two groups in terms of platelet count, vascular risk factors, stroke etiology, and lesion location (Table 1).

**Table 1:** Demographic and clinical characteristics of the samples under study

Baseline characteristics	Non‐PSD (*n* = 125)	PSD (*n* = 60)	*p* value
Demographic characteristics			
Female, *n* (%)	36 (28.8)	29 (48.3)	0.009
Age (years), mean ± *SD*	63 ± 19	62 ± 10	0.535
Educational years, median (IQR)	5 (0–8)	3 (0–6)	0.227
BMI (kg/m^2^), mean ± *SD*	24.31 ± 3.52	25.38 ± 3.63	0.202
Marital Status, married, *n* (%)	125 (100.0)	59 (98.3)	0.324
Vascular risk factors			
Hypertension, *n* (%)	91 (72.8)	45 (75.0)	0.751
Diabetes mellitus, *n* (%)	34 (27.2)	15 (25.0)	0.751
Coronary heart disease, *n* (%)	11 (8.8)	3 (5.0)	0.554
Hyperlipidemia, *n* (%)	12 (9.6)	11 (18.3)	0.092
History of stroke, *n* (%)	13 (10.4)	5 (8.3)	0.657
Current smoking, *n* (%)	46 (36.8)	16 (26.7)	0.172
Current drinking, *n* (%)	49 (39.2)	16 (26.7)	0.095
Lesion location, *n* (%)			
Left hemisphere	43 (34.4)	15 (25.0)	0.062
Right hemisphere	35 (28.0)	24 (40.0)	
Brainstem	21(16.8)	13 (21.7)	
Cerebellum	10 (8.0)	0 (0.0)	
Other	16 (12.8)	8 (13.3)	
TOAST classification, *n* (%)			
LA	114 (91.2)	52 (86.7)	0.235
CE	7 (5.6)	2 (3.3)	
SA	2 (1.6)	3 (5.0)	
SOE	0 (0.0)	1 (1.7)	
SUE	2 (1.6)	2 (3.3)	
Neuropsychological function			
NIHSS score, median (IQR)	3 (1–4)	4 (2–6)	<0.001
MMSE score, median (IQR)	24 (22–28)	23 (20–27)	0.017
mRS (IQR)	1 (1–2)	2 (1–3)	0.001
BI (IQR)	97 (100–100)	88 (80–100)	<0.001
Platelet count, mean ± *SD*	219 ± 60	216 ± 59	0.821
MPV(fl), mean ± *SD*	8.5 ± 1.6	9.3 ± 1.8	0.002

Note

BMI: body mass index; CE: cardio embolism; LA: large‐artery atherosclerosis; NIHSS: National Institutes of Health Stroke Scale; mRS: modified Rankin Scale; MMSE: mini mental state examination; BI: modified Barthel Index; MPV: mean platelet volume; IQR: interquartile range; *SD*: standard deviation; PSD: Poststroke depression; SA: small‐artery occlusion Lacunar; SOE: stroke of other determined etiology; SUE: stroke of other undetermined etiology.

### 3.2 The association between MPV and PSD

As demonstrated in Table 2, there were significant differences between PSD and non‐PSD in MPV tertiles of patients (*p = *0.005). Moreover, in the lowest tertile (≤7.7 fl), PSD group had a lower proportion of patients than that in non‐PSD group (*p* = 0.002), while in the highest tertile (≥9.1 fl), PSD group had a higher proportion of patients than that in non‐PSD group (*p = *0.010).

**Table 2:** MPV tertiles of patients

	PSD patients (*n* = 60)	Non‐PSD patient (*n* = 125)	*p* value
MPV, *n* (% of total population)			0.005
Tertile 1 (≤7.7 fl)	13 (21.7%)	51 (40.8%)	0.002
Tertile 2 (7.8–9.0 fl)	18 (30.0%)	42 (33.6%)	0.624
Tertile 3 (≥9.1 fl)	29 (48.3%)	32 (25.6%)	0.010

Note

MPV: mean platelet volume; PSD: poststroke depression.

As shown in Table 3, in the binary logistic regression analysis, tertile 2 was taken as reference and PSD presence was taken as a dependent variable, the high tertile of MPV levels (≥9.1 fl) was independently correlated with PSD (OR 2.762, 95% CI 1.138–6.702, *p* = 0.025). Moreover, the NIHSS score on admission as well as BI score at 1 month were significantly correlated with PSD (OR 1.237, 95% CI 1.027–1.490, *p* = 0.025, and OR 0.938, 95% CI 0.887–0.993, *p* = 0.027). Furthermore, gender and hyperlipidemia were significantly correlated with PSD (OR 2.457, 95% CI 1.058–5.704, *p = *0.036, and OR 3.604, 95% CI 1.252–10.379, *p* = 0.018).

**Table 3:** Multivariate logistic model of the clinical determinants of poststroke depression

Variables	OR (95%CI)	*p* value
MPV		0.009
Tertile 1		0.481
Tertile 3	2.762 (1.138–6.702)	0.025
Female	2.457 (1.058–5.704)	0.036
Hyperlipidemia	3.604 (1.252–10.379)	0.018
Current drinking		0.746
Lesion location		0.214
NIHSS	1.237 (1.027–1.490)	0.025
BI	0.938 (0.887–0.993)	0.027
mRS		0.603
MMSE score		0.277

Note

MPV: mean platelet volume; OR: odds ratio; CI: confidence interval; NIHSS: National Institutes of Health Stroke Scale; mRS: modified Rankin Scale; MMSE: mini mental state examination; BI: modified Barthel Index.

## 4 DISCUSSION

To our best knowledge, this was the first report to examine the association between MPV levels and the presence of PSD. Our results indicated that high MPV levels were independently correlated with PSD.

In this study, 32.4% of patients developed PSD at 1 month after stroke, the prevalence is similar to previous researches (Allan et al., 2013; Hackett & Pickles, 2014; Hackett, Yapa, Parag, & Anderson, 2005). We found that female gender was significantly correlated with PSD, which was consistent with earlier findings (Guiraud et al., 2016; Mazure, Weinberger, Pittman, Sibon, & Swendsen, 2014; Zhang, Zhao, Fang, Wang, & Zhou, 2017). Our results also showed that physical disability and stroke severity were correlated with PSD, which were in line with previous researches (Eriksen, Gay, & Lerdal, 2016; Ko et al., 2018; Kutlubaev & Hackett, 2014; Sarfo et al., 2017). In addition, we found that hyperlipidemia was associated with PSD. This finding was in accord with previous studies (Armstrong et al., 2017; Lim et al., 2017). However, there was no significant association between PSD and other clinical characteristics, such as lesion location (Guiraud, et al., 2016; Hama et al., 2007; Metoki et al., 2016), although among some of previous studies the results about lesion location remained controversial.

Our results are in line with another study that demonstrated increased MPV levels were associated with depression in a large population (Canan et al., 2012). Likewise, elevated MPV levels were also found in patients with fibromyalgia syndrome as well as bipolar disorder (Vural et al., 2015; Yildirim, Solmaz, Akgol, & Ersoy, 2016). Furthermore, previous studies showed that MPV levels reduced significantly after antidepressant treatment (Ataoglu & Canan, 2009; Demircan et al., 2016).

The relationship between MPV and depression remains not completely understood. However, several mechanisms may explain the depression in patients with higher MPV levels. The regulation of the platelet activity might explain the association between MPV and depression. As a routinely available measure of platelet size at admission, MPV can be considered as a biological indicator of platelet activity (Chu, et al., 2010), which was correlated with depression (Musselman, et al., 1996; Shimbo et al., 2002). This accorded well with one study based on a large population, which reported that elevated MPV levels were associated with major depression (Canan, et al., 2012). Meanwhile, it has been shown that escitalopram effect of inhibiting reuptake of serotonin occurs in neurons as well as platelets, reducing platelet reactivity (Atar et al., 2006). Besides, as mentioned earlier, patients with depression exhibited normalization of platelet activation and significant reduction in MPV levels after escitalopram treatment (Ataoglu & Canan, 2009), indicating that MPV may play a vital role in pathophysiological process of depression. Thus, considering its role in ischemic stroke as well as depression, MPV may participate in the development of PSD.

Our study still has some limitations. First, patients with serious conditions or aphasia were excluded from our study, which may reduce the real PSD prevalence. Second, only MPV levels in the baseline period was measured, which might make us fail to reveal the dynamic change in MPV levels during the development of PSD. Finally, the application of our conclusion may be limited in those minor stroke subjects, for most of the patients with a higher NIHSS score have been excluded.

## 5 CONCLUSION

In conclusion, despite these limitations, the findings of this study remained important and demonstrated that high levels of MPV on admission were significantly associated with the development of PSD. High MPV levels could be considered as an independent prognostic marker of PSD. These findings suggest a potential role of MPV in the pathophysiology of PSD and indicate that patients with acute ischemic stroke should be monitored for high MPV levels. In future, multicenter and randomized controlled trials are critical to determine the causal relationship between MPV levels and PSD.

## CONFLICT OF INTEREST

None declared.
